# Protective Effect of Lusianthridin on Hemin-Induced Low-Density Lipoprotein Oxidation

**DOI:** 10.3390/ph14060567

**Published:** 2021-06-14

**Authors:** Su Wutyi Thant, Noppawan Phumala Morales, Visarut Buranasudja, Boonchoo Sritularak, Rataya Luechapudiporn

**Affiliations:** 1 Pharmaceutical Sciences and Technology Program, Faculty of Pharmaceutical Sciences, Chulalongkorn University, Bangkok 10330, Thailand; suwutyithantt@gmail.com; 2 Department of Pharmacology, Faculty of Sciences, Mahidol University, Bangkok 10400, Thailand; noppawan.phu@mahidol.ac.th; 3 Department of Pharmacology and Physiology, Faculty of Pharmaceutical Sciences, Chulalongkorn University, Bangkok 10330, Thailand; visarut.b@pharm.chula.ac.th; 4 Department of Pharmacognosy and Pharmaceutical Botany, Faculty of Pharmaceutical Sciences, Chulalongkorn University, Bangkok 10330, Thailand; Boonchoo.Sr@chula.ac.th; 5 Natural Products for Ageing and Chronic Diseases Research Unit, Chulalongkorn University, Bangkok 10330, Thailand

**Keywords:** lusianthridin, hemin, oxidation of low-density lipoprotein, cholesteryl linoleate, cholesteryl arachidinate, foam cell formation

## Abstract

Oxidation of low-density lipoprotein (LDL) plays a crucial role in the pathogenesis of atherosclerosis. Hemin (iron (III)-protoporphyrin IX) is a degradation product of hemoglobin that can be found in thalassemia patients. Hemin is a strong oxidant that can cause LDL oxidation and contributes to atherosclerosis in thalassemia patients. Lusianthridin from *Dendrobium venustrum* is a phenolic compound that possesses antioxidant activity. Hence, lusianthridin could be a promising compound to be used against hemin-induced oxidative stress. The major goal of this study is to evaluate the protective effect of lusianthridin on hemin-induced low-density lipoprotein oxidation (he-oxLDL). Here, various concentrations of lusianthridin (0.25, 0.5, 1, and 2 µM) were preincubated with LDL for 30 min, then 5 µM of hemin was added to initiate the oxidation, and oxidative parameters were measured at various times of incubation (0, 1, 3, 6, 12, 24 h). Lipid peroxidation of LDL was measured by thiobarbituric reactive substance (TBARs) assay and relative electrophoretic mobility (REM). The lipid composition of LDL was analyzed by using reverse-phase HPLC. Foam cell formation with he-oxLDL in RAW 264.7 macrophage cells was detected by Oil Red O staining. The results indicated that lusianthridin could inhibit TBARs formation, decrease REM, decrease oxidized lipid products, as well as preserve the level of cholesteryl arachidonate and cholesteryl linoleate. Moreover, He-oxLDL incubated with lusianthridin for 24 h can reduce the foam cell formation in RAW 264.7 macrophage cells. Taken together, lusianthridin could be a potential agent to be used to prevent atherosclerosis in thalassemia patients.

## 1. Introduction

Atherosclerosis is a chronic disease of the arterial wall. Oxidation of low-density lipoprotein (LDL) plays a significant role in the initiation and development of atherosclerosis [1]. LDL can be oxidized by transition metals, such as iron [2] and hemin [3]. Metal iron-induced oxidation is essential in lipid peroxidation and protein oxidations of lipoproteins [4]. Hemin (iron III -protoporphyrin IX), the degradation product of hemoglobin oxidation, appears in the plasma because of intravascular hemolysis [5]. Hemin can be detected in the serum of β-thalassemia/hemoglobin (β-thal/HbE) patients, then vascular complications and early atherosclerosis commonly occurred in these patients [6]. The ferric component of hemin penetrates the hydrophobic core of LDL [7] and causes LDL oxidation. Exposure of LDL to hemin produces lipid peroxidation products and modification in the apolipoprotein of LDL. The oxidized form of LDL becomes recognized by scavenger receptors of macrophages and increased cholesterol inside the cell, generating the macrophage-derived foam cells, which are the hallmark of atherosclerosis [8].

Lipid composition in the core of LDL particles consists of cholesterol esters (CE) and triglyceride (TG). The structure of cholesterol ester contains cholesterol and polyunsaturated fatty acids, such as arachidonic acid, linoleic acid, oleic acid, and palmitic acid. The most abundant cholesteryl ester in LDL is cholesteryl linoleate (CL), a major target of lipid peroxidation [9]. Once the free radical attaches to the LDL, it can cause oxidation of polyunsaturated fatty acids and increase the formation of malondialdehyde (MDA). Hemin can be used as a potent inducer for LDL oxidation [8]. In hemin-induced LDL oxidation (he-oxLDL), the level of cholesteryl linoleate decreased dramatically, leading to an increase in the oxidized products of cholesteryl esters that may be involved in the development of atherosclerosis [10].

*Dendrobium* is one of the largest and most important genera in the family Orchidaceae [11]. They can be found in Asian countries, and nearly 150 species have been found in Thailand [12]. They are widely used in traditional Chinese medicine to reduce fever and hyperglycemia [13]. *Dendrobium* species produce several bioactive components, including phenanthrenes, bibenzyls, alkaloids, fluorenones, sesquiterpenes, coumarins, and polysaccharides. These compounds have been reported to possess various pharmacological activities, such as anti-inflammatory, antiplatelet aggregation, and antioxidant activities [12]. The antioxidant and free radical scavenging activities of *Dendrobium* species have been studied [14]. Lusianthridin (LST), a phenanthrene compound extracted from *Dendrobium venustum*, exhibited antioxidant activity determined by DPPH radical scavenging activity, with an IC_50_ of 21.40 ± 0.61 µg/mL and FRAP value of 1071.46 ± 46.58 mg Trolox/g dry wt. [13]. So, LST is a promising antioxidant compound for the prevention of LDL oxidation from an oxidizing agent. However, the effect of LST on LDL oxidation induced by hemin has not been investigated yet. Therefore, the aims of this study were mainly to evaluate the protective effect of lusianthridin on lipid composition in hemin-induced LDL oxidation and on foam cell formation in RAW 264.7 macrophages.

## 2. Results

### 2.1. Effect of Lusianthridin on TBARs Formation

**Figure 1:**
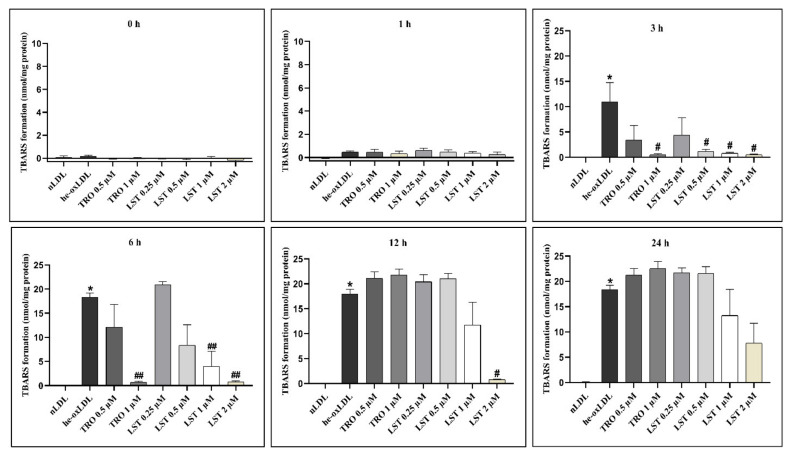
Effect of lusianthridin on TBARs formation in hemin-induced LDL oxidation. LDLs were preincubated with LST or Trolox for 30 min, then hemin was added to induce LDL oxidation. After 24 h treatment, TBARs formations were determined at various time points with TBARs assay. The data are presented as mean ± SEMs (*n* = 5); * *p* < 0.05 *vs*. nLDL, ^#^ *p* < 0.05 and ^##^ *p* < 0.001 *vs*. he-oxLDL.

The formation of TBARs in hemin-induced LDL is shown in Figure 1. Hemin rapidly decreased α-tocopherol content in LDL. The α-tocopherol content was undetectable within 1 h (data not shown). TBARs formation was gradually increased after depletion of α-tocopherol. TBARs level of he-oxLDL at 0, 1, and 3 h were 0.17 ± 0.09, 0.46 ± 0.08, and 10.92 ± 3.85 nmol/mg protein, respectively. The TBARs formation reached a plateau level at 6 h (18.25 ± 0.92 nmol/mg protein). Although LST did not restore α-tocopherol content, the high concentrations of LST (1 and 2 µM) significantly inhibited TBARs formation at 3 h, approximately 22.8 and 54.7%, respectively. The inhibitory activity of LST lasted up to 24 h. However, Trolox significantly inhibited TBARs formation for at least only 6 h. The inhibitory activity of Trolox cannot be observed at 12 and 24 h. Therefore, our results suggested that LST could possess a stronger magnitude in antioxidant activity and could have a longer duration of inhibitory activity against lipid peroxidation than Trolox.

### 2.2. Effect of Lusianthridin on Relative Electrophoretic Mobility

**Figure 2:**
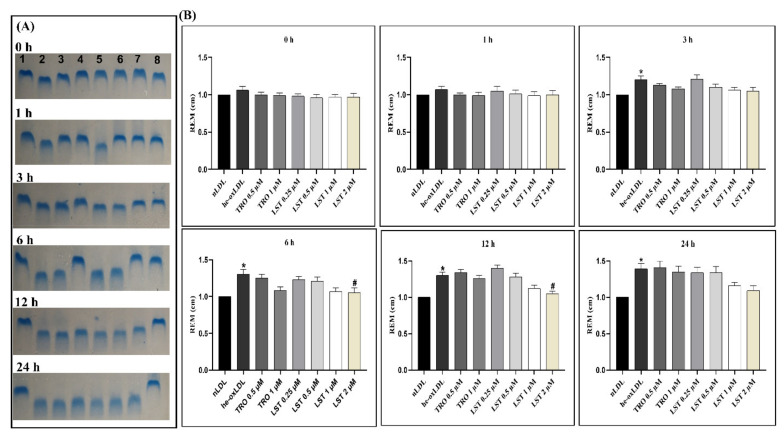
Effect of lusianthridin on relative electrophoretic mobility (REM) in hemin-induced LDL oxidation at various times of incubation. (**A**) Representative REM in agarose electrophoresis. Lane 1: nLDL, lane 2: he-oxLDL, lane 3: trolox 0.5 µM, lane 4: trolox 1 µM, lane 5: LST 0.25 µM, lane 6: LST 0.5 µM, lane 7: LST 1 µM, lane 8: LST 2 µM; (**B**) REM data as mean ± SEMs (*n* = 5), * *p* < 0.05 *vs*. nLDL; ^#^ *p* < 0.05 *vs*. he-oxLDL.

Oxidation of apolipoprotein in LDL is demonstrated by increasing relative electrophoretic mobility (REM) (Figure 2). REM of he-oxLDL at 3, 6, 12, and 24 h was significantly increased to 1.2 ± 0.05, 1.3 ± 0.07, 1.3 ± 0.05, and 1.39 ± 0.08, respectively. These results indicated that hemin could progressively induce protein oxidation in LDL. Trolox protected protein oxidation at 1 µM, and the protective effect was long-lasting, at least 6 h. Although the low concentration of LST at 0.25 and 0.5µM could not prevent protein oxidation, the higher concentration 1 and 2 µM can reduce REM. The duration of the protective effect was comparable to Trolox.

### 2.3. Effect of Lusianthridin on Lipid Level

**Figure 3:**
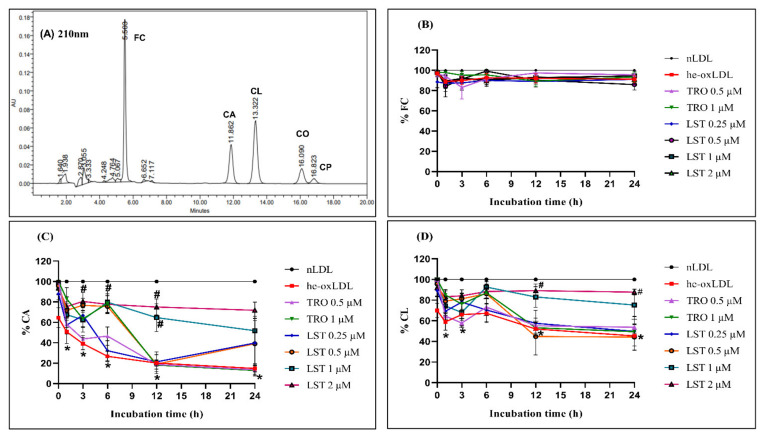
Effects of LST on lipid composition in hemin-induced LDL oxidation for 24 h. (**A**) Representative chromatogram of lipid composition detected at 210 nm; (**B**) % free cholesterol FC; (**C**) % cholesteryl arachidonate, CA; (**D**) % cholesteryl linoleate, CL; data were presented as mean ± SEMs (*n* = 4); * *p* < 0.05 *vs*. nLDL; ^#^ *p* < 0.05 *vs*. he-oxLDL.

Decreasing cholesteryl esters of unsaturated fatty acids is a marker of lipid peroxidation in lipoproteins. The CA and CL levels of he-oxLDL decreased to 30 ± 9.2% and 75.6 ± 8.1% at 24 h, respectively (Figure 3). LST at 0.25 and 0.5 µM could not protect against the reduction of CA and CL. However, higher concentration significantly attenuated the reduction. The retaining levels of CA at 24 h were 51.85 ± 12.58 and 71.87 ± 7.15%, and those of CL were 75.26 ± 9.6 and 87.72 ± 2.54% for LST at 1 and 2 µM, respectively. In the presence of Trolox 1 µM, both CA and CL levels were not decreased significantly until 6 h. Then, the level progressively decreased to 12.87 ± 3.39% and 49.41 ± 6.06% at 24 h.

**Figure 4:**
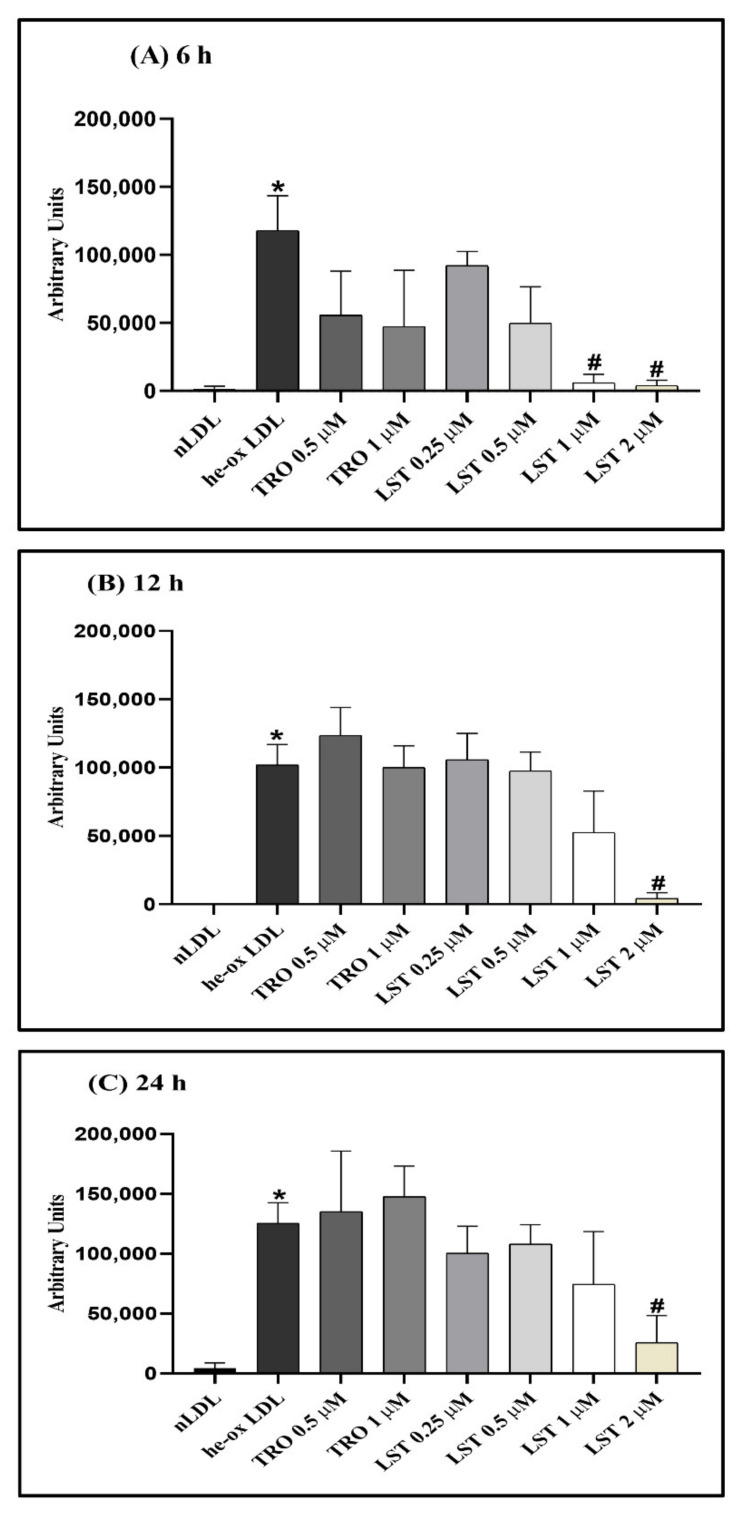
Effect of lusianthridin on oxidized lipid products detected at 234 nm: (**A**) at 6 h, (**B**) at 12 h, and (**C**) at 24 h in hemin-induced LDL oxidation. Data were presented as mean ± SEMs (*n* = 4); * *p* < 0.05 *vs*. nLDL; ^#^ *p* < 0.05 *vs*. he-oxLDL.

Corresponding to the decreasing of CA and CL, the oxidized lipids were produced. The oxidized lipid products in he-oxLDL were detectable from 6 h (Figure 4). Thus, LST reduced oxidized lipid products in a concentration-dependent manner, whereas Trolox could not reduce oxidized lipid products.

### 2.4. Effect of Lusianthridin on RAW 264.7 Macrophage Cell Viability

**Figure 5:**
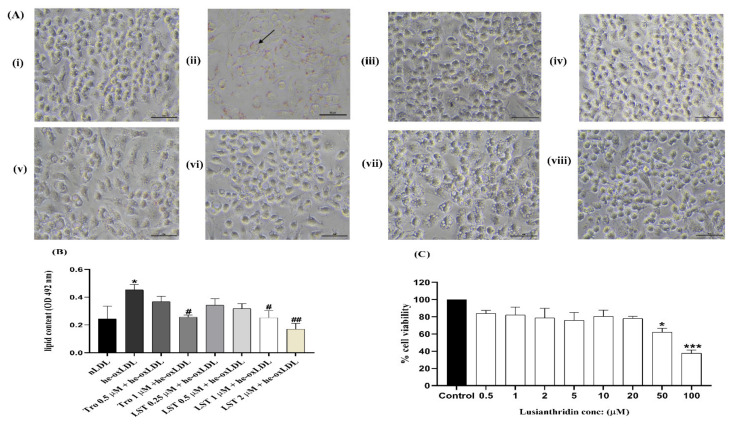
(**A**) Representative Oil Red O photographs of RAW 264.7 macrophage cells (40 × magnification) (i) nLDL, (ii) he-oxLDL, (iii) TRO 0.5 µM, (iv) TRO 1 µM, (v) LST 0.25 µM, (vi) LST 0.5 µM, (vii) LST 1 µM, (viii) LST 2 µM. (**B**) Quantitation of lipid content at 492 nm. Data were presented as mean ± SEMs (*n* = 4); * *p* < 0.05 *vs*. nLDL; ^#^ *p* < 0.05 and ^##^ *p* < 0.0001 *vs*. he-oxLDL. (**C**) Effect of lusianthridin on RAW 264.7 macrophage cell viability. * *p* < 0.05 and ^***^ *p* < 0.0001 *vs*. control.

RAW 264.7 macrophage cells were used to investigate the effects of LST on he-oxLDL-induced formation of foam cells. Prior to conducting the foam cell formation assay, the safety profile of LST needed to be established. RAW cells were incubated with various concentrations of LST for 24 h, and the viability of cells was evaluated with MTT assay. The effect of LST on cell viability of RAW 264.7 cells is shown in Figure 5C. The cell viability was significantly decreased at 50 and 100 µM, but the lower concentrations were not significantly different from the control. The cytotoxic effect of LST was observed at concentrations of 50 and 100 µM, much higher than the antioxidant concentration. This result indicated that LST at concentrations lower than 20 µM had no cytotoxic effect on RAW 264.7 macrophage cells, and 0.25, 0.5, 1, and 2 µM of LST can be used for foam cell formation assay.

### 2.5. Effect of Lusianthridin on Foam Cell Formation in RAW 264.7 Macrophages

The RAW 264.7 macrophage cells exposed to he-oxLDL showed a foam cell phenotype, which is characterized by lipid droplet formation (Figure 5A). In addition, lipid content in cells incubated with he-oxLDL was significantly different from nLDL (Figure 5B). Corresponding with the antioxidative activity of Trolox and LST, exposure of he-oxLDL treated with 1 µM Trolox, and 1 and 2 µM LST reduced the foam cell formation and decreased lipid content compared to he-oxLDL. However, a low concentration of Trolox and LST could not reduce foam cell formation.

## 3. Discussion

Modification of LDL is one of the crucial factors in the initiation of atherosclerosis. Therefore, antioxidants become the potential treatment for the prevention of LDL oxidation. Plants which contain phenanthrene derivative have many biological activities, including antioxidant activity. *Dendrobium* species are among the largest and most important genera in the family Orchidaceae, including phenanthrene derivatives that showed antioxidant activity. According to the result of this study, lusianthridin, a phenanthrene derivative from *Dendrobium venustum*, becomes a potential compound for preventing LDL oxidation induced by hemin.

Hemin is a potent oxidizing agent that induces oxidation in both lipid and protein components of LDL. Because of its lipophilicity, hemin readily binds to LDL and releases ferric iron (Fe^3+^) to the hydrophobic core of LDL. Iron exerts toxicity through a series of reactions with reactive oxygen species, including highly toxic hydroxyl radical. This hydroxyl radical rapidly reacts with polyunsaturated fatty acids (PUFAs) in the core cholesteryl esters of LDL and initiates a chain reaction process. Here, the he-oxLDL system was used to evaluate the protective effect of lusianthridin on LDL oxidation [15].

Hemin can increase TBARs formation and also modify the apolipoprotein of LDL. Increasing in TBARs formation might be due to the depletion of cholesteryl linoleate (CL) [16], which is the most abundant and significant target of the lipid peroxidation process [9]. When treating he-oxLDL with lusianthridin, lusianthridin can inhibit both TBARs formation and protein oxidation of LDL in a concentration-dependent fashion. It is more potent than Trolox, the positive control (Figure 1 and Figure 2). According to TBARs and REM results, lusianthridin inhibited TBARs formation induced by hemin by reaching the maximum percent inhibition of 95.2% at 2 µM until 12 h. From these findings, we could estimate that lusianthridin has a protective effect in LDL oxidation until 12 h.

Moreover, hemin also causes changes in lipid composition of the LDL core, especially in cholesteryl arachidonate (CA) and cholesteryl linoleate (CL), which comprise polyunsaturated fatty acids (PUFA) that cause more oxidation than monounsaturated and saturated fatty acids [17] and also increased oxidized lipid products. Decreasing CA and CL might be due to the hydrolysis of the ester bond and the oxidized lipid products that were produced by lipid peroxidation of CA and CL [15]. Furthermore, hemin causes undetectable α-tocopherol. According to entrapping free radical intermediates in this process, this effect might be due to α-tocopherol suppressing aldehyde formation, which could occur by free radical reactions as a secondary process of lipid peroxidation [18]. The oxidative markers in this in vitro he-oxLDL have characteristics like those for LDL in β-thal/HbE patients, which were shown by an increase in TBARs, depletion of α-tocopherol, decreasing CA and CL levels, and increasing oxidized lipid products [9]. In this study, lusianthridin preserved both CL and CA levels in a concentration-dependent manner compared with Trolox (Figure 3). Therefore, it seems that lusianthridin might inhibit the hydrolysis of cholesteryl esters. Moreover, lusianthridin also decreased oxidative lipid products in this study (Figure 4). It inhibited TBARs formation and protein oxidation, which means that it could reduce lipid peroxidation induced by hemin. From our findings, we concluded that lusianthridin possesses a protective effect on hemin-induced LDL oxidation.

Additionally, modification of apolipoprotein by LDL oxidation may lead to foam cell formation. Foam cell formation is the accumulation of lipids, and it is the early stage of atherosclerosis [19]. Oxidized LDL causes several proatherogenic effects, such as endothelial cells and monocyte stimulation, which cause increased inflammatory cytokine, chemokine, adhesion molecules, and stimulation of scavenger receptors of macrophage LOX-1, CD36, and SR-A1 [19,20]. Modification of apolipoprotein by oxidized LDL is unrecognized by the lipoprotein receptor (LDLR), whereas it becomes engulfed by scavenger receptors of macrophages (LOX-1, CD36, and SR-A1). Therefore, it is believed that foam cell formation may be caused by the changes in LDLR, LOX-1, CD36, and SR-A1. In this study, foam cell formation occurred and it might be due to the effect of lipid peroxidation of LDL oxidation. Lipid peroxidation causes engulfment by scavenger receptors of macrophages (LOX-1, CD 36, and SR-A1) and causes foam cell formation. This study showed that lusianthridin reduced foam cell formation (Figure 5) and it can be assumed that lusianthridin possibly inhibits the engulfment of he-oxLDL by scavenger receptors of RAW 264.7 macrophage cells.

**Figure 6:**
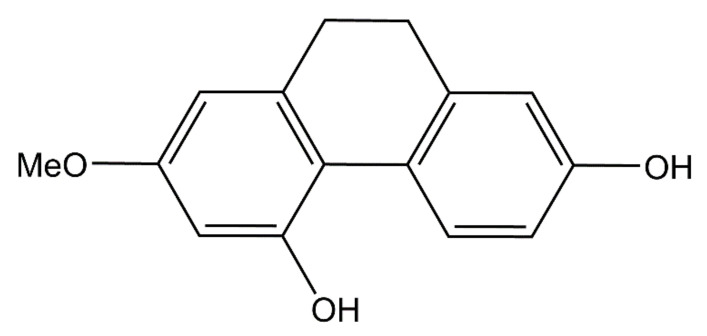
Structure of lusianthridin.

In this study, it could be assumed that the protective effect of lusianthridin on hemin-induced LDL oxidation might come from the presence of a phenanthrenes group and phenolic compound. Lusianthridin used in this study was extracted from *Dendrobium venustum* and is a plant phenolic compound in the stilbenoid group (phenanthrenes). Phenanthrenes derivative from *Ephemerantha lonchophylla* showed that phenanthrenes had the inhibitory activity of lipoprotein oxidation [21] and phenanthrene from *Brassica rapa* showed the inhibitory of LDL oxidation [22]. Therefore, the presence of a hydroxyl group on the structure of lusianthridin (Figure 6) might be essential for antioxidant activity [23] and this activity might be reduced by oxidation of LDL. Furthermore, the phenolic compound reduces Fe^2+^ to Fe^3+^, which includes the Fenton reaction. Fe^3+^ generates the toxic hydroxyl radical, which reacts with cholesteryl ester in hydrophobic core lipoprotein and initiates lipid peroxidation [15]. Hence, it could be possible that lusianthridin inhibited lipid peroxidation of LDL induced by hemin due to the reduction of Fe^3+^. Moreover, a reduction in foam cell formation might be due to the inhibition of the lipid peroxidation effect of lusianthridin. In summary, lusianthridin could protect hemin-induced low-density lipoprotein oxidation and could be a potential agent to be used in clinic to prevent atherosclerosis in thalassemia patients.

## 4. Materials and Methods

### 4.1. Materials

The following chemicals were obtained from Sigma Chemical Co. (St. Louis, MO, USA): ethylene diamine tetraacetic acid disodium dehydrate (Na_2_EDTA), potassium bromide (KBr), potassium chloride, disodium phosphate, potassium dihydrogen phosphate, butylated hydroxyl toluene (BHT), sodium dodecyl sulfate (SDS), trichloroacetic acid (TCA), thiobarbituric acid, and dimethyl sulfoxide (DMSO). Sodium chloride (NaCl) was obtained from Merck (Darmstadt, Germany). Acetonitrile, isopropanol, hexane, and butanol were obtained from RCI Lab Scan Co., Ltd. (Bangkok, Thailand). Agarose powder, Coomassie blue solution, and Tris-acetate-EDTA (TAE) buffer were obtained from Bio-Rad Laboratories (California, USA). DMEM powder, fetal bovine serum, penicillin, and streptomycin were obtained from Gibco (New York, USA). Lusianthridin (LST) was isolated from *Dendrobium venustum* [12] and its purity was evaluated using NMR spectroscopy.

### 4.2. Preparation of LDL

The study was performed in human low-density lipoprotein (LDL) obtained from healthy volunteers age 18 to 30 years, with no prior or ongoing medical conditions, non-smokers, non-alcoholics, not donating blood in the last one month, and not taking any medications at least 2 weeks before participating in the study. This study was approved by the Institutional Review Board, Faculty of Medicine, Chulalongkorn University (COA No. 493/2020, approval date on 20 April 2020).

LDL was separated from the pooled plasma of three overnight-fasting healthy volunteers. Young women tend to have lower LDL levels, with higher HDL levels than young men [24]. Although the lipid compositions in LDL are not different between genders, only women were recruited to participate in this study to minimize the variation among each independent experiment. Firstly, 30 mL of each blood sample were collected in a tube containing Na_2_EDTA (final concentration: 1 mg/mL blood) as an anticoagulant. The pooled plasma was separated by centrifugation (Falcon:6300) 2454× *g* for 15 min at 4 °C, and stored at −80 °C until LDL separation. LDL was separated by sequential density gradient ultracentrifugation method using Hitachi CP100 NX (Tokyo, Japan) ultracentrifuge with P100AT2 Fixed angle rotor (himac, Tokyo, Japan). Plasma was adjusted to the density of LDL (1.019–1.063 g/mL) with salt solution containing KBr, NaCl, and EDTA, and then centrifuged at 289,000× *g* at 16 °C to obtain LDL. The isolated LDL was dialyzed with 10 mM phosphate buffer saline (pH 7.4) overnight to remove EDTA and salts before induction of LDL oxidation by hemin. The protein concentration of LDL was determined by using a nanodrop UV–visible spectrophotometer.

### 4.3. Hemin-Induced LDL Oxidation

LDL (400 µg/mL) was preincubated with the various concentrations (0.25, 0.5, 1, and 2 µM) of LST for 30 min in a shaking incubator at 37 °C, then hemin (5 µM) was added to induce oxidation of LDL for 24 h. In addition, 0.5 and 1 µM of Trolox were used as a positive control. Each concentration of LST and Trolox was performed in duplicate in 5 independent experiments. The aliquots were collected at various incubation times (0, 1, 3, 6, 12, 24 h). Then, the reaction was terminated by adding 100 µM EDTA and 5 mM BHT.

### 4.4. Formation of Thiobarbituric Reactive Substance (TBARs)

TBARs was used as an oxidative marker of the lipid peroxidation process. The oxidative breakdown of unsaturated fatty acids reacted with thiobarbituric acid (TBA) and produced a pink-colored adduct with fluorescent adduct. TBARs determination was modified from Akasawa T. [25]; briefly, 100 µL of he-oxLDL was mixed well with 100 µL of 10% TCA, 50 µL of 8% SDS, and 150 µL of 6% TBA and heated at 95 °C for 1 h. Then, TBARs were extracted by 400 µL of butanol, then measured using a spectrofluorometer with excitation and emission wavelength at 515 nm and 553 nm, respectively. Tetramethoxyproane was used as standard. The data were presented as nanomoles of TBARs per milligram LDL protein [9].

### 4.5. Relative Electrophoretic Mobility (REM) of LDL

The negative charge modification in LDL was determined by agarose gel electrophoresis. REM of LDL was used as a marker of apolipoprotein oxidation. Native LDL (nLDL) and hemin-oxidized LDL (he-oxLDL) at various times of incubation were run on 1% agarose gel in TAE buffer at constant voltage (70 V) for 45 min. The protein on the gel was stained with Coomassie blue solution for 30 min and then gently washed with water [26]. The distance from origin was measured and expressed as REM.

### 4.6. Determination of the Level of Lipid

Lipid composition and α-tocopherol were analyzed by reverse-phase HPLC using a UV–vis detector at 210 nm for free cholesterol and cholesteryl ester, 234 nm for oxidized lipid products, and fluorescence detector with excitation 295 nm and emission 370 nm for α-tocopherol.

The lipid in he-oxLDL was extracted by adding ice-cold methanol and hexane, and then vortex for about 30 s and 1 min. The hexane layer was dried under nitrogen and then redissolved in acetonitrile. After that, the samples were injected into a Hypersil BDS C-18 stainless steel column (5 µm; 4.6 × 250 mm) (Thermo Scientific, California, USA) with the a mobile phase of 46% acetonitrile: 53% isopropanol: 3% water. The flow rate was 1 mL/min and the temperature of the column was controlled at 50 °C. The standard solution of free cholesterol, cholesteryl arachidonate, cholesteryl linoleate, and α-tocopherol was prepared and the amount of lipid composition can be calculated from the chromatogram [27].

### 4.7. Cell Culture

RAW 264.7 macrophage cell line was purchased from American Type Culture Collection (ATCC; Virginia, USA). RAW cells were cultured in high glucose DMEM medium supplemented with 10% fetal bovine serum, penicillin (100 U/mL), and streptomycin (100 µg/mL). Cells were maintained at 37 °C in a humidified atmosphere, 5% CO_2_ and 95% air, and grown to 70–80% confluence.

### 4.8. Cell Viability Assay

Cell viability of RAW 264.7 macrophage cells was determined by using traditional MTT assay. This method is based on the conversion of yellow tetrazolium MTT into a purple-colored formazan product by mitochondrial enzymes of viable cells [28]. After experimental treatments, cells were incubated with MTT solution (0.5 mg/mL in serum-free-DMEM medium) for 3 h, and then 200 μL of DMSO was added to dissolve formazan crystals. The absorbance was then measured at 570 nm by a CLARIOstar microplate reader ( BMG Labtech, Ortenberg, Germany) and the cell viability was expressed related to the vehicle control (0.5% DMSO).

### 4.9. Foam Cell Formation Detection

The preparation of 1 mg/mL of nLDL, he-oxLDL, or he-oxLDL with LST or Trolox was incubated for 24 h in a shaking incubator at 37 °C. RAW 264.7 macrophage cells (1 × 10^5^ cells) in 24-well plates were then treated with 100 µg/mL of nLDL or he-oxLDL for 24 h. The cells were fixed with 4% formaldehyde for 15 min and then stained with Oil Red O solution for 6 min at 37 °C. The cells were washed with 60% isopropanol, and then gently washed with PBS. The staining cells were observed under the inverted microscope (Nikon) [29]. Lipid content in RAW 264.7 macrophage cells was estimated by determining the content of Oil Red O in the stained cell. The cell was further extracted with 100% isopropanol and the absorbance was quantified at 492 nm by a microplate reader.

### 4.10. Statistical Analysis

All experimental results were expressed as means ± standard error of means. Differences between groups were analyzed by One-way ANOVA, followed by Tukey’s post hoc test using SPSS software version 22.0.. *P* values less than 0.05 were accepted as statistical significance. 

## 5. Conclusions

This study demonstrated that lusianthridin, a phenanthrene from *Dendrobium venustum*, protected LDL oxidation induced by hemin, and lusianthridin also showed the potential protective effect in foam cell formation. Further detailed studies of lusianthridin could be performed in animal models of β-thalassemia or atherosclerosis and it will be beneficial in preventing disease-related LDL oxidation.

## Data Availability

Data is contained within the article.
